# Mining User Reviews From Hypertension Management Mobile Health Apps to Explore Factors Influencing User Satisfaction and Their Asymmetry: Comparative Study

**DOI:** 10.2196/55199

**Published:** 2024-03-28

**Authors:** Yunfan He, Wei Zhu, Tong Wang, Han Chen, Junyi Xin, Yongcheng Liu, Jianbo Lei, Jun Liang

**Affiliations:** 1 Center for Health Policy Studies School of Public Health Zhejiang University Hangzhou China; 2 Department of Cardiology Second Affiliated Hospital, College of Medicine Zhejiang University Hangzhou China; 3 Cardiovascular Key Laboratory of Zhejiang Province Hangzhou China; 4 School of Health and Life Sciences University of Health and Rehabilitation Sciences Qingdao China; 5 School of Basic Medical Sciences Shandong University Jinan China; 6 Qingdao Hospital University of Health and Rehabilitation Sciences (Qingdao Municipal Hospital) Qingdao China; 7 Department of Cardiology Second Affiliated Hospital, College of Medicine Zhejiang University Hangzhou China; 8 School of Information Engineering Hangzhou Medical College Hangzhou China; 9 Netease Group Hangzhou China; 10 Clinical Research Center Affiliated Hospital of Southwest Medical University Luzhou China; 11 The First Affiliated Hospital Hainan Medical University Haikou China; 12 Center for Medical Informatics Health Science Center Peking University Beijing China; 13 Department of AI and IT Second Affiliated Hospital, School of Medicine Zhejiang University Hangzhou China; 14 Key Laboratory of Cancer Prevention and Intervention, China National Ministry of Education, School of Medicine Zhejiang University Hangzhou China; 15 School of Medical Technology and Information Engineering Zhejiang Chinese Medical University Hangzhou China

**Keywords:** hypertension management, mobile health, topic modeling, satisfaction, 2-factor model, comparative study

## Abstract

**Background:**

Hypertension significantly impacts the well-being and health of individuals globally. Hypertension management apps (HMAs) have been shown to assist patients in controlling blood pressure (BP), with their efficacy validated in clinical trials. However, the utilization of HMAs continues to be suboptimal. Presently, there is a dearth of real-world research based on big data and exploratory mining that compares Chinese and American HMAs.

**Objective:**

This study aims to systematically gather HMAs and their user reviews from both China and the United States. Subsequently, using data mining techniques, the study aims to compare the user experience, satisfaction levels, influencing factors, and asymmetry between Chinese and American users of HMAs. In addition, the study seeks to assess the disparities in satisfaction and its determinants while delving into the asymmetry of these factors.

**Methods:**

The study sourced HMAs and user reviews from 10 prominent Chinese and American app stores globally. Using the latent Dirichlet allocation (LDA) topic model, the research identified various topics within user reviews. Subsequently, the Tobit model was used to investigate the impact and distinctions of each topic on user satisfaction. The Wald test was applied to analyze differences in effects across various factors.

**Results:**

We examined a total of 261 HMAs along with their associated user reviews, amounting to 116,686 reviews in total. In terms of quantity and overall satisfaction levels, Chinese HMAs (n=91) and corresponding reviews (n=16,561) were notably fewer compared with their American counterparts (n=220 HMAs and n=100,125 reviews). The overall satisfaction rate among HMA users was 75.22% (87,773/116,686), with Chinese HMAs demonstrating a higher satisfaction rate (13,866/16,561, 83.73%) compared with that for American HMAs (73,907/100,125, 73.81%). Chinese users primarily focus on reliability (2165/16,561, 13.07%) and measurement accuracy (2091/16,561, 12.63%) when considering HMAs, whereas American users prioritize BP tracking (17,285/100,125, 17.26%) and data synchronization (12,837/100,125, 12.82%). Seven factors (easy to use: *P*<.001; measurement accuracy: *P*<.001; compatibility: *P*<.001; cost: *P*<.001; heart rate detection function: *P*=.02; blood pressure tracking function: *P*<.001; and interface design: *P*=.01) significantly influenced the positive deviation (PD) of Chinese HMA user satisfaction, while 8 factors (easy to use: *P*<.001; reliability: *P*<.001; measurement accuracy: *P*<.001; compatibility: *P*<.001; cost: *P*<.001; interface design: *P*<.001; real-time: *P*<.001; and data privacy: *P*=.001) affected the negative deviation (ND). Notably, BP tracking had the greatest effect on PD (β=.354, *P*<.001), while cost had the most significant impact on ND (β=3.703, *P*<.001). All 12 factors (easy to use: *P*<.001; blood pressure tracking function: *P*<.001; data synchronization: *P*<.001; blood pressure management effect: *P*<.001; heart rate detection function: *P*<.001; data sharing: *P*<.001; reliability: *P*<.001; compatibility: *P*<.001; interface design: *P*<.001; advertisement distribution: *P*<.001; measurement accuracy: *P*<.001; and cost: *P*<.001) significantly influenced the PD and ND of American HMA user satisfaction. Notably, BP tracking had the greatest effect on PD (β=0.312, *P*<.001), while data synchronization had the most significant impact on ND (β=2.662, *P*<.001). In addition, the influencing factors of PD and ND in user satisfaction of HMA in China and the United States are different.

**Conclusions:**

User satisfaction factors varied significantly between different countries, showing considerable asymmetry. For Chinese HMA users, ease of use and interface design emerged as motivational factors, while factors such as cost, measurement accuracy, and compatibility primarily contributed to user dissatisfaction. For American HMA users, motivational factors were ease of use, BP tracking, BP management effect, interface design, measurement accuracy, and cost. Moreover, users expect features such as data sharing, synchronization, software reliability, compatibility, heart rate detection, and nonintrusive advertisement distribution. Tailored experience plans should be devised for different user groups in various countries to address these diverse preferences and requirements.

## Introduction

The global prevalence of hypertension is on the rise. Hypertension management apps (HMAs) serve as convenient tools for effectively managing blood pressure (BP). These apps enhance users’ awareness of self-management, dietary and exercise habits, and medication adherence through features such as BP tracking, dietary guidance, exercise monitoring, educational resources, and medication reminders. The ultimate aim is to achieve effective BP control. Their effectiveness has been demonstrated in experimental settings. Globally, the number of patients with hypertension surged from 648 million in 1990 to 1.278 billion in 2019, marking a prevalence of 33% [1]. HMAs represent digital health tools with the potential for effectively controlling BP [2-9]. Their usability [10,11] and effectiveness [12] have been demonstrated in randomized controlled trials. In a systematic review and meta-analysis of 18 randomized controlled trials, Han et al [13] found that HMAs could significantly reduce BP and improve BP control rates. Moreover, within the framework of the current value-based medical policy, a hospital’s treatment efficacy relies not only on its in-hospital care but also on postdischarge patient attention and management. As a collaborative tool for out-of-hospital medical services, HMAs can assist patients in sustaining the effects of in-hospital treatments, thereby reducing the rate of hospital readmissions. McManus et al [14] demonstrated that compared with traditional nursing interventions, apps yield superior BP control effects within a year and incur lower incremental costs. However, in practical settings, many HMAs have been developed without adhering to evidence-based medicine [15] and lacked clinical certification before marketing [16], potentially posing adverse effects on hypertension management [17,18].

Although HMAs show significant BP management effects in controlled environments, their real-world outcomes are often unsatisfactory. Both user engagement and retention rates are low, with 62% of mobile health (mHealth) apps having fewer than 1000 monthly active users [19] and only 6.6% of patients with hypertension continuing to use HMAs [20]. These situations directly correlate with user satisfaction [21-24]. Therefore, enhancing user satisfaction can significantly improve the effectiveness of the app [25]. According to the Food and Drug Administration (FDA), in real-world scenarios, health information technology (HIT) is integrated into a complex sociotechnical system, and its actual impact is influenced by 4 primary factors aside from the product itself: people (whether they are involved or not), technologies (including HIT hardware and software), processes (the workflow of health care delivery), and organization (the procedure for HIT installation and configuration). Among these factors, external environmental elements (such as the policies and cultures of different countries) play a significant role [26]. The actual impacts of HMAs can vary significantly among user groups in different countries. Therefore, it is essential to explore and compare the satisfaction levels and influencing factors of user groups across various countries. This comparative analysis can enable targeted efforts to enhance the practical effectiveness of HMAs.

Traditional studies on user satisfaction of HMAs primarily consist of qualitative analyses [27,28]. However, these studies often lack breadth and depth, characterized by limitations such as small sample sizes, susceptibility to adverse observer effects and recall bias, and limited generalizability of conclusions. Furthermore, these studies often overlook the impact of external environmental factors and do not compare or analyze the satisfaction levels of user groups across different countries. For instance, Li et al [29] conducted semistructured interviews with 13 English-speaking patients with hypertension to explore their surface-level satisfaction with HMAs and analyze factors contributing to dissatisfaction. Kang and Park [30] developed an English HMA grounded on clinical guidelines for hypertension management. They used the modified Morisky Scale to assess perceived usefulness and satisfaction with this app among 38 patients diagnosed with hypertension. Although qualitative analyses allow for a deep exploration of individual attitudes, they have limitations such as a restricted number of apps that can be analyzed [31,32], a small sample size confined to a specific region [33], high research costs, and potential biases in conclusions [34].

Research on user satisfaction with HMAs lacks both quantitative studies driven by large volumes of user-generated content and in-depth exploration of the factors influencing satisfaction, particularly in terms of asymmetry. In addition, there is a notable absence of comparative analyses of user groups across different countries. Plante et al [35] manually annotated and summarized English user reviews of an HMA (Instant BP) from an online app store, discovering that users expressed greater satisfaction with apps yielding lower measurement results. Similarly, Wang et al [36] and Nuo et al [37] conducted quantitative analyses of user reviews for weight and sleep management apps to investigate user satisfaction and influencing factors. However, none of these studies conducted an in-depth evaluation of variations in satisfaction and influencing factors among user groups across different countries. The renowned Herzberg 2-factor theory [38] in management suggests that the factors influencing user satisfaction exhibit asymmetry and can be categorized into motivational factors that enhance satisfaction and hygienic factors that mitigate dissatisfaction. Using large-sample data and incorporating the asymmetry of factors impacting app user satisfaction, while also considering variations among user groups in different countries, can effectively mitigate errors in constructing explanatory models. This approach enhances the predictability and generalizability of the model [39].

Therefore, this study adopts the 2-factor model and uses unsupervised clustering algorithms to quantitatively analyze user reviews of HMAs from major Chinese and American app stores globally. By considering the macro usage environment, the study aims to extract and compare the primary opinions of Chinese and American users, assess differences in satisfaction and its influencing factors, and explore the asymmetry within these factors.

## Methods

### Informed Consent and Study Approval Statement

All data used in this study were sourced from publicly accessible internet mobile app stores, encompassing app information and user reviews. Hence, this study does not entail any medical ethics concerns.

### Data Collection

#### HMA Identification

**Figure 1:**
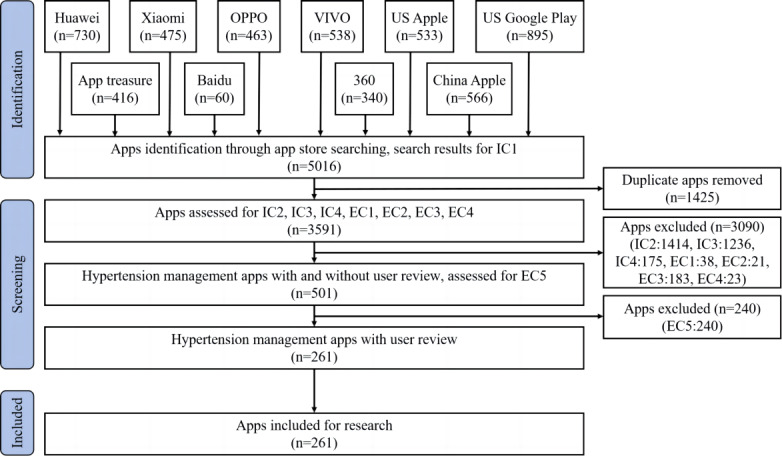
Flowchart of the hypertension management app screening process; IC: inclusion criteria; EC: exclusion criteria.

In this comparative study, to conduct a comprehensive review of the primary HMAs, we consulted previous studies [36,37] and identified 10 widely used Chinese and American app stores from the 2 major mobile phone platforms (iOS [Apple Inc.] and Android [Google LLC]). These include 8 Chinese platforms (China Apple, Huawei, Xiaomi, OPPO, VIVO, Baidu, 360, and Application Treasure) and 2 American platforms (US Apple App Store and US Google Play Store). App stores in different countries can only access user data from their respective regions. Therefore, in April 2023, we conducted searches across the aforementioned 10 Chinese and American app stores using keywords such as “hypertension,” “high blood pressure,” “blood pressure management,” and “blood pressure recording.” We retrieved a total of 5016 apps, out of which 3591 remained after deduplication (Table S1 in Multimedia Appendix 1). Following guidelines from previous studies [40], we formulated detailed inclusion and exclusion criteria (Table S2 in Multimedia Appendix 1), which were independently screened by 2 researchers (YFH and J Liang). Both researchers underwent standardized training before the screening process, resulting in high consistency in their screening results (κ=0.84). Any discrepancies between the researchers were resolved through arbitration by another cardiovascular clinical expert (WZ). The specific screening process, following the PRISMA (Preferred Reporting Items for Systematic Reviews and Meta-Analyses) 2020 guidelines [41], is depicted in Figure 1.

#### User Review Collection

All 10 Chinese and American app platforms offer user ratings and review features, enabling users to provide quantitative ratings and qualitative descriptions of their satisfaction with apps. These ratings typically range from 1 (very poor) to 5 (very good). We used Python scripts (Python Foundation) and the Qimai mobile app data analysis platform [42] to extract all quantitative ratings and qualitative user reviews of the included HMAs. As of April 23, 2023, we collected a total of 295,927 quantitative ratings and qualitative user reviews, comprising 250,193 reviews from American users and 45,734 reviews from Chinese users. The proportion of Chinese reviews and downloads (16,561/24,204,832, 0.07%) is similar to that of American reviews and downloads (100,125/148,869,181, 0.07%).

### User Review Preprocessing

The presence of false and meaningless user reviews in the app store data significantly impacted the topic mining of user reviews and interfered with the assessment of user satisfaction with HMAs. Hence, we conducted preprocessing on the user reviews with the following steps:

Removing data containing only ratings without accompanying user reviews.

Excluding user reviews posted on bot accounts, using the tweetbotornot package in R [43]. This resulted in the exclusion of 53,431 American and 16,940 Chinese reviews.

Eliminating duplicate reviews, blank values, non-Chinese or English reviews, garbled characters, and reviews deemed meaningless. This process led to the exclusion of 80,829 American and 9697 Chinese reviews.

**Figure 2:**
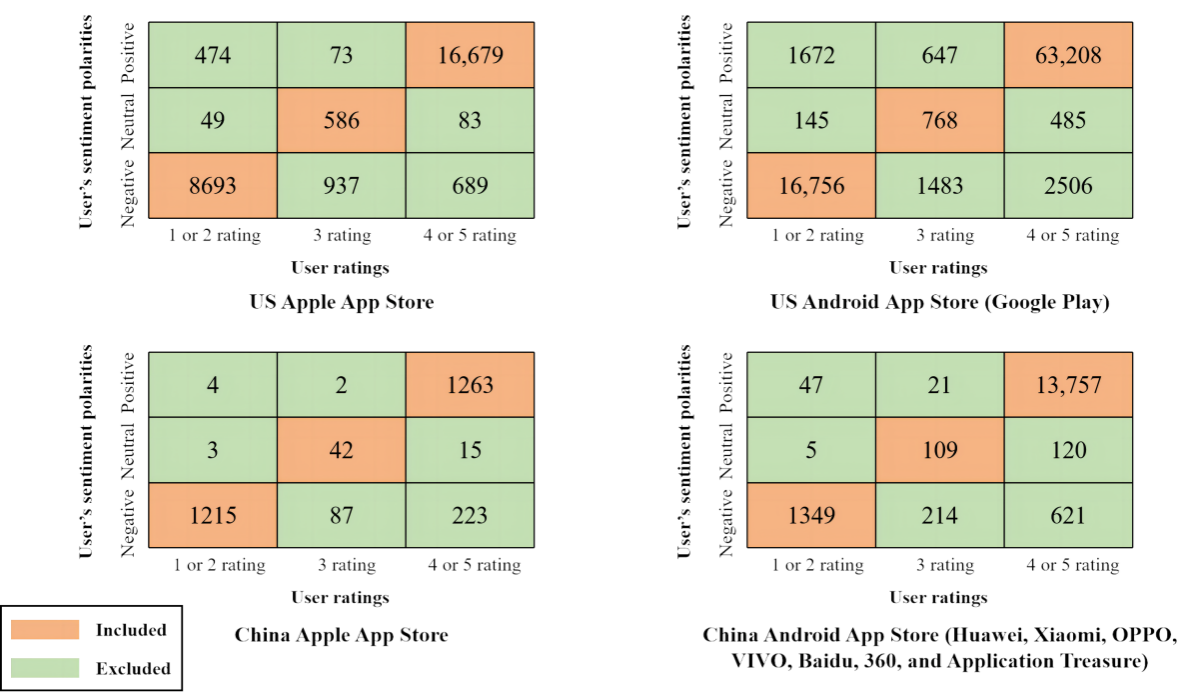
Matching between user’s sentiment polarities (rows) and user ratings (columns).

Following this, we used the sentiment knowledge–enhanced pretraining algorithm [44] to calculate the emotional polarity of each user review, categorized into negative, neutral, and positive sentiments. Furthermore, contradictory data showing inconsistencies between user ratings and reviews were eliminated [45], resulting in the exclusion of 9243 American and 1362 Chinese reviews. Specifically, we removed data with user ratings of 1 or 2 points that lacked negative emotional polarities in user reviews, data with user ratings of 3 points that lacked neutral emotional polarities in user reviews, and data with user ratings of 4 or 5 points that missed positive emotional polarities in user reviews (Figure 2). Finally, we uniformly labeled different data with the same concept (Table S3 in Multimedia Appendix 1). Following the initial data preprocessing steps, a total of 124,425 user reviews were included in the LDA model for topic modeling, comprising 106,690 American reviews and 17,735 Chinese reviews.

### Data Analysis

#### Overview

We used natural language processing technology and the LDA topic model to extract the main topics from both Chinese and American user reviews. Subsequently, following the 2-factor model, we constructed a Tobit model to analyze the correlation between different topics and user satisfaction. Finally, we used the Wald test to analyze the differences in the impact of each topic on user satisfaction.

#### LDA Topic Modeling

LDA is a widely used probability-based topic modeling algorithm [46]. It is known for its ease of operation, high efficiency, and positive impact on topic clustering and prediction accuracy [47]. As LDA is primarily a language model rather than a classification prediction model, perplexity serves as a common and effective indicator for evaluating the quality of the language model, rather than sensitivity. To efficiently and accurately extract the primary opinions and topics from Chinese and American user reviews, we used LDA, which is a 3-level hierarchical Bayesian model. LDA calculates the distribution probabilities of words and topics, enabling the clustering of latent semantic structures within user reviews to summarize the main topics.

For the LDA model input, we used the word segmentation set and specified the number of Chinese and American review topics, along with manually induced topics. Consequently, we executed the LDA topic modeling process as follows: Initially, we used the Jieba [48] and NLTK packages [49] in Python to segment the Chinese and American reviews, respectively. We then used stop-word lists compiled from Baidu, Harbin Institute of Technology, Sichuan University Machine Intelligence Laboratory, and standard Chinese and English stop words [50]. These stop-word lists were applied to delete stop words, including numbers, punctuation marks, emoticons, and blank values, from both Chinese and English word segmentations. Subsequently, any blank reviews were removed after the stop words had been eliminated. In addition, we conducted morphological restoration of English word segmentation. Subsequently, based on the perplexity curve and actual clustering effect [51], we determined that 11 topics were optimal for Chinese reviews and 12 topics for American reviews (Figures S1–S4 in Multimedia Appendix 1). Furthermore, we used the Gensim package [52] in Python to construct the LDA topic models for both Chinese and American reviews. Two researchers (YFH and J Liang) independently summarized and named the topics for each keyword set in the Chinese and American review topic models, respectively. Any discrepancies were resolved through arbitration by a third expert (WZ). Finally, the topics for each user review were determined based on their distribution probabilities across all topics generated by the LDA model [53].

#### Statistical Analysis

To delve deeper into the factors influencing user satisfaction with HMAs, in line with the 2-factor theory [38], we introduced 2 variables: positive deviation (PD) and negative deviation (ND) [54]. These variables are defined by the disparity between the user’s individual rating of HMAs and the overall rating displayed in the app store. They serve to indicate the discrepancy between the user’s personal rating and the average rating. PD and ND are mutually exclusive if a user’s deviation is positive and the ND value is 0, and vice versa. To simplify the calculation, we applied absolute value processing to ND. Consequently, the higher the ND value, the greater the degree of dissatisfaction. Both PD and ND values ranged between 0 and 4, calculated based on the difference between the user rating range and the comprehensive rating range of the app store. We opted for the Tobit model [55], known for its effectiveness in handling limited dependent variables, to assess the factors influencing user satisfaction. The probability distribution of each LDA topic model for each user review served as the independent variable in our analysis. The PD and ND of each user review were used as dependent variables, and the specific model was defined in Multimedia Appendix 1. Finally, we used the Wald test [54] to perform a difference test on the absolute values of the PD and ND model parameters, aiming to identify the asymmetry of the factors influencing user satisfaction. Given that the Tobit model uses the maximum likelihood method to fit the best parameters, conventional methods cannot be applied for the robustness analysis of the model results. In addition, the exogenous variable in this study, namely, the distribution probability of each topic in user reviews, encompasses all factors affecting user satisfaction, thereby mitigating endogeneity concerns. Model establishment and data analysis were conducted using Stata 16.0 (StataCorp) [56], with a significance level set at a 2-sided *P*<.05 for the difference test.

## Results

### Chinese and American HMA User Review Topics

We initially retrieved 5016 related apps from both Chinese and American app stores, yielding a total of 295,927 original reviews in both languages. Following screening and data preprocessing, we identified 261 HMAs with user reviews. Among these, 41 (15.7%) were exclusively available in Chinese, 170 (65.1%) were exclusively available in American, and 50 (19.2%) were available in both app stores. Ultimately, 116,686 user reviews were included in the analysis, with 100,125 (85.81%) in English and 16,561 (14.19%) in Chinese. Among these reviews, 87,773 (including 73,907 American reviews and 13,866 Chinese reviews) were rated 4 stars and above, resulting in an overall satisfaction rate of 75.22%: 73,907/100,125 (73.81%) for American apps and 13,866/16,561 (83.73%) for Chinese apps.

The results of LDA modeling revealed differences between Chinese and American reviews. In Chinese reviews, the number of reviews related to software reliability (2165/16,561, 13.07%) and measurement accuracy (2091/16,561, 12.63%) substantially exceeded other topics. Conversely, specific hypertension management functions such as BP tracking (17,285/100,125, 17.26%) and data synchronization (12,837/100,125, 12.82%) substantially outnumbered other topics in American reviews.

The LDA modeling revealed 11 topics in the Chinese reviews, with 5 (ease of use: *P*<.001 in both PD and ND; interface design: *P*=.01 in PD and *P*<.001 in ND; measurement accuracy: *P*<.001 in both PD and ND; compatibility: *P*<.001 in both PD and ND; and cost: *P*<.001 in both PD and ND) of them being significant (including 2 motivational factors and 3 hygienic factors). By contrast, the American reviews yielded 12 topics, all of which were significant (easy to use: *P*<.001 in both PD and ND; blood pressure tracking function: *P*<.001 in both PD and ND; data synchronization: *P*<.001 in both PD and ND; blood pressure management effect: *P*<.001 in both PD and ND; heart rate detection function: *P*<.001 in both PD and ND; data sharing: *P*<.001 in both PD and ND; reliability: *P*<.001 in both PD and ND; compatibility: *P*<.001 in both PD and ND; interface design: *P*<.001 in both PD and ND; advertisement distribution: *P*<.001 in both PD and ND; measurement accuracy: *P*<.001 in both PD and ND; cost: *P*<.001 in both PD and ND), comprising 6 motivational factors and 6 hygienic factors (Tables 1 and 2; Tables S4–S7 in Multimedia Appendix 1).

**Table 1:** Topics and keywords of Chinese reviews formulated by latent Dirichlet allocation topic modeling (N=16,561).

Topics	Keywords	Reviews, n (%)
Topic 1: Easy to use	simple, convenient, easy to use, practical, rapid, recommended, easy to operate	6863 (41.44)
Topic 2: Reliability	healthy, quality, comprehensive, supportive, stable, professional, normal	2165 (13.07)
Topic 3: Measurement accuracy	measurement, accurate, collect, accuracy, test, data, inaccurate	2091 (12.63)
Topic 4: Attitude (positive)	very good, like, recommend, every day, awesome, useful, look forward to	1850 (11.17)
Topic 5: Compatibility	version, download, try, blood pressure monitor, Apple, connection, platform	878 (5.30)
Topic 6: Cost	fee, subscription, free, payment, upgrade, refund, paid	653 (3.94)
Topic 7: Heart rate detection function	heart rate, detection, value, body, monitor, watch, indicator	597 (3.60)
Topic 8: Blood pressure tracking function	blood pressure, function, record, tool, data, share, form	502 (3.03)
Topic 9: Interface design	updated, special, interface, clear, design, components, good-looking	471 (2.84)
Topic 10: Real-time	trial, view, status, anytime, anywhere, patient, daily	299 (1.81)
Topic 11: Data privacy	account, personal, information, security, management, privacy, licensing	192 (1.16)

**Table 2:** Topics and keywords of American reviews formulated by latent Dirichlet allocation topic modeling (N=100,125).

Topics	Keywords	Reviews, n (%)
Topic 1: Easy to use	easy, love, simple, handy, utility, recommend, worth	34,443 (34.40)
Topic 2: Blood pressure tracking function	blood, pressure, monitor, track, check, record, measure	17,285 (17.26)
Topic 3: Data synchronization	time, phone, update, synchronization, connect, omron, data	12,837 (12.82)
Topic 4: Blood pressure management effect	good, help, care, body, hypertension, maintain, condition	11,881 (11.87)
Topic 5: Heart rate detection function	heart, rate, pulse, check, measure, test, monitor	7065 (7.06)
Topic 6: Data sharing	data, email, export, send, share, access, require	4465 (4.46)
Topic 7: Reliability	quality, bad, screen, complete, fake, uninstalling, bug	3793 (3.79)
Topic 8: Compatibility	version, iphone, upgrade, fine, android, fail, reinstall	2586 (2.58)
Topic 9: Interface design	user, wonderful, friendly, interface, experience, unit, type	2390 (2.39)
Topic 10: Advertisement distribution	advertisement, download, watch, garbage, click, poor, difficult	1599 (1.60)
Topic 11: Measurement accuracy	accurate, cuff, feel, offer, result, manual, actual	1199 (1.20)
Topic 12: Cost	cost, fee, free, money, afford, pay, count	582 (0.58)

### Factors Affecting Chinese and American HMA User Satisfaction

We computed the variance inflation factor of the PD and ND models for both Chinese and American reviews to assess multicollinearity among the independent variables. The regression variance inflation factors of all independent variables in the 4 models were found to be <5, indicating the absence of multicollinearity-related issues [22] (Tables S8-S11 in Multimedia Appendix 1). Furthermore, in Chinese reviews, the topic “attitude (positive)” pertains solely to users’ positive attitudes and does not encompass opinions regarding the functions and utility of apps. Therefore, it was not included in the Tobit regression model.

In Table 3, model 1 displays the PD model results for Chinese reviews. With the exception of reliability (*P*=.56), real-time (*P*=.21), and data privacy (*P*=.52), the other 7 topics (easy to use: *P*<.001; measurement accuracy: *P*<.001; compatibility: *P*<.001; cost: *P*<.001; heart rate detection function: *P*=.02; blood pressure tracking function: *P*<.001; and interface design: *P*=.01) discussed by users significantly influenced the PD of user satisfaction. Among them, the factors with the most substantial positive and negative effects were the BP tracking function (β=.354, *P*<.001) and cost (β=–.232, *P*<.001), respectively. Model 2 presents the ND model results for Chinese reviews. Except for the heart rate detection (*P*=.64) and BP tracking (*P*=.14) functions, the other 8 topics (easy to use: *P*<.001; reliability: *P*<.001; measurement accuracy: *P*<.001; compatibility: *P*<.001; cost: *P*<.001; interface design: *P*<.001; real-time: *P*<.001; and data privacy: *P*=.001) significantly impacted user satisfaction NDs. The factors with the most substantial positive and negative effects were cost (β=3.703, *P*<.001) and interface design (β=–1.619, *P*<.001), respectively. In Table 4, model 3 presents the PD model results for American reviews. All 12 topics (easy to use: *P*<.001; blood pressure tracking function: *P*<.001; data synchronization: *P*<.001; blood pressure management effect: *P*<.001; heart rate detection function: *P*<.001; data sharing: *P*<.001; reliability: *P*<.001; compatibility: *P*<.001; interface design: *P*<.001; advertisement distribution: *P*<.001; measurement accuracy: *P*<.001; and cost: *P*<.001) included in the model significantly influenced the user satisfaction PD. Among them, the factors with the most significant positive and negative effects were the BP tracking function (β=.312, *P*<.001) and data synchronization (β=–.593, *P*<.001), respectively. Model 4 displays the ND model results for American reviews. All 12 topics (easy to use: *P*<.001; blood pressure tracking function: *P*<.001; data synchronization: *P*<.001; blood pressure management effect: *P*<.001; heart rate detection function: *P*<.001; data sharing: *P*<.001; reliability: *P*<.001; compatibility: *P*<.001; interface design: *P*<.001; advertisement distribution: *P*<.001; measurement accuracy: *P*<.001; and cost: *P*<.001) significantly influenced the user satisfaction ND. Data synchronization (β=2.662, *P*<.001) had the greatest positive effect, while the BP management effect (β=–2.035, *P*<.001) had the most substantial negative effect.

**Table 3:** Determinant factors for rating deviations (Chinese reviews^a^).

Influencing factor	Model 1^b^				Model 2^c^		
	β (95% CI)	SE	*P* value	β (95% CI)		SE	*P* value
Topic 1: Easy to use	0.080 (0.043 to 0.117)	0.019	<.001	–1.575 (–1.792 to –1.358)		0.111	<.001
Topic 2: Reliability	0.013 (–0.030 to 0.055)	0.022	.56	–0.692 (–0.937 to –0.448)		0.125	<.001
Topic 3: Measurement accuracy	–0.181 (–0.224 to –0.138)	0.022	<.001	2.789 (2.564 to 3.014)		0.115	<.001
Topic 5: Compatibility	–0.219 (–0.275 to –0.163)	0.029	<.001	3.170 (2.906 to 3.434)		0.135	<.001
Topic 6: Cost	–0.232 (–0.293 to –0.171)	0.031	<.001	3.703 (3.424 to 3.981)		0.142	<.001
Topic 7: Heart rate detection function	–0.073 (–0.132 to –0.014)	0.030	.02	0.077 (–0.250 to 0.403)		0.167	.64
Topic 8: Blood pressure tracking function	0.354 (0.298 to 0.411)	0.029	<.001	–0.239 (–0.556 to 0.078)		0.162	.14
Topic 9: Interface design	0.082 (0.019 to 0.144)	0.032	.01	–1.619 (–1.934 to –1.304)		0.161	<.001
Topic 10: Real-time	–0.047 (–0.121 to 0.026)	0.038	.21	1.459 (1.091 to 1.826)		0.188	<.001
Topic 11: Data privacy	–0.028 (–0.113 to 0.057)	0.043	.52	0.761 (0.315 to 1.208)		0.228	.001

^a^In Chinese reviews, the topic labeled “topic attitude (positive)” pertains solely to users’ positive attitudes and does not encompass opinions regarding the functions and utility of apps. Therefore, it was not included in the Tobit regression model

^b^Positive deviations: The maximum likelihood estimate of model 1 was –16521.383.

^c^Negative deviations: The maximum likelihood estimate of model 2 was –12843.104.

**Table 4:** Determinant factors for rating deviations (American reviews).

Influencing factor	Model 3^a^				Model 4^b^			
	β (95% CI)	SE	*P* value	β (95% CI)		SE	*P* value	
Topic 1: Easy to use	0.068 (0.058 to 0.078)	0.005	<.001	–0.946 (–0.995 to –0.898)		0.025	<.001	
Topic 2: Blood pressure tracking function	0.312 (0.301 to 0.323)	0.006	<.001	–1.139 (–1.195 to –1.083)		0.029	<.001	
Topic 3: Data synchronization	–0.593 (–0.606 to –0.581)	0.006	<.001	2.662 (2.612 to 2.711)		0.025	<.001	
Topic 4: Blood pressure management effect	0.247 (0.235 to 0.260)	0.006	<.001	–2.035 (–2.100 to –1.970)		0.033	<.001	
Topic 5: Heart rate detection function	–0.201 (–0.214 to –0.188)	0.007	<.001	0.807 (0.750 to 0.863)		0.029	<.001	
Topic 6: Data sharing	–0.150 (–0.165 to –0.135)	0.008	<.001	0.707 (0.642 to 0.771)		0.033	<.001	
Topic 7: Reliability	–0.269 (–0.283 to –0.255)	0.007	<.001	1.356 (1.295 to 1.417)		0.031	<.001	
Topic 8: Compatibility	–0.233 (–0.252 to –0.214)	0.010	<.001	1.467 (1.394 to 1.540)		0.037	<.001	
Topic 9: Interface design	0.155 (0.138 to 0.171)	0.008	<.001	–1.235 (–1.324 to –1.146)		0.046	<.001	
Topic 10: Advertisement distribution	–0.577 (–0.599 to –0.555)	0.011	<.001	2.644 (2.565 to 2.723)		0.040	<.001	
Topic 11: Measurement accuracy	0.039 (0.019 to 0.058)	0.010	<.001	–0.597 (–0.694 to –0.499)		0.050	<.001	
Topic 12: Cost	0.217 (0.192 to 0.243)	0.013	<.001	–0.664 (–0.920 to –0.408)		0.130	<.001	

^a^Positive deviations: The maximum likelihood estimate of model 3 was –81779.181.

^b^Negative deviations: The maximum likelihood estimate of model 4 was –102855.05.

### Motivational and Hygienic Factor Asymmetry

The effect asymmetry results are presented in Tables S12 and S13 in Multimedia Appendix 1. In models 1 and 2, with the exception of the heart rate detection and BP tracking functions, the remaining 8 factors exhibited significant differences in asymmetric effect (easy to use: *P*<.001; reliability: *P*<.001; measurement accuracy: *P*<.001; compatibility: *P*<.001; cost: *P*<.001; interface design: *P*<.001; real-time: *P*<.001; and data privacy: *P*=.001). In models 3 and 4, all 12 influencing factors demonstrated significant differences (easy to use: *P*<.001; blood pressure tracking function: *P*<.001; data synchronization: *P*<.001; blood pressure management effect: *P*<.001; heart rate detection function: *P*<.001; data sharing: *P*<.001; reliability: *P*<.001; compatibility: *P*<.001; interface design: *P*<.001; advertisement distribution: *P*<.001; measurement accuracy: *P*<.001; and cost: *P*<.001). Consequently, all factors exhibited a significant asymmetric effect on user satisfaction with Chinese (easy to use: *P*<.001; reliability: *P*<.001; measurement accuracy: *P*<.001; compatibility: *P*<.001; cost: *P*<.001; interface design: *P*<.001; real-time: *P*<.001; and data privacy: *P*=.001) and American HMAs (easy to use: *P*<.001; blood pressure tracking function: *P*<.001; data synchronization: *P*<.001; blood pressure management effect: *P*<.001; heart rate detection function: *P*<.001; data sharing: *P*<.001; reliability: *P*<.001; compatibility: *P*<.001; interface design: *P*<.001; advertisement distribution: *P*<.001; measurement accuracy: *P*<.001; and cost: *P*=.001).

Regarding the influencing factors of user satisfaction with Chinese HMAs, both ease of use (*P*<.001 for both) and interface design (*P*=.01 and *P*<.001, respectively) had significant positive or negative effects on the PD or ND models, respectively. In addition, the effects on the 2 models were significantly different (*P*<.001). Similarly, measurement accuracy (*P*<.001 for both), compatibility (*P*<.001 for both), and cost (*P*<.001 for both) had a significant negative or positive impact on the PD or ND model, with a significant difference in the effect of the 2 models (*P*<.001).

Regarding the factors influencing user satisfaction with American HMAs, ease of use (*P*<.001 for both), BP tracking function (*P*<.001 for both), BP management effect (*P*<.001 for both), interface design (*P*<.001 for both), measurement accuracy (*P*<.001 for both), and cost (*P*<.001 for both) all exhibited significant positive or negative effects on the PD or ND model. Moreover, there was a significant difference in the effect on the 2 models (*P*<.001). Data synchronization (*P*<.001 for both), heart rate detection function (*P*<.001 for both), data sharing (*P*<.001 for both), reliability (*P*<.001 for both), compatibility (*P*<.001 for both), and advertisement distribution (*P*<.001 for both) each had a significant negative or positive impact on the PD or ND model. Furthermore, the impact on the 2 models was significantly different (*P*<.001).

## Discussion

### Principal Findings

To the best of our knowledge, this study represents the first quantitative analysis of user satisfaction, influencing factors, and asymmetry of HMA-related factors based on user reviews. By encompassing a large number of apps and user samples, our research achieved high credibility at a low cost, rendering the findings highly generalizable. Furthermore, leveraging the sociotechnical model widely used in HIT evaluation, we conducted a comparative analysis of Chinese and American HMAs, elucidating differences in user satisfaction and influencing factors between Chinese and American user groups. Consequently, we offer targeted improvement suggestions based on our findings. Although the numbers of Chinese HMAs and reviews were lower than those of American apps, user satisfaction was higher with Chinese HMAs. Furthermore, the main factors influencing user satisfaction and dissatisfaction with Chinese HMAs were the BP tracking function and cost, respectively. Conversely, the main factors affecting user satisfaction and dissatisfaction with American HMAs were the BP tracking function and data synchronization, respectively. Regarding the asymmetry of influencing factors, all factors exhibited significantly different effects on user satisfaction and dissatisfaction. Moreover, there were notable disparities in the motivational and hygienic factors between Chinese and American HMA users.

### Differences in the Use of Chinese and American HMAs

Globally, users are distributed unevenly, with considerably lower usage of Chinese HMAs compared with American HMAs. As HMAs represent a typical form of digital health software, their usage status can be effectively analyzed using sociotechnical models [25], which are widely used in HIT. External environmental factors, such as medical policies, diagnosis and treatment methods, and payment methods in different countries, serve as major determinants of HIT. These factors may explain the significant disparities in the use of Chinese and American HMA. Regarding medical policies and treatment methods, Chinese patients tend to rely more on hospital-based doctors, while American patients often prefer active health management guided by family doctors. In China, a 3-level accreditation system for general hospitals has been implemented, and diagnosis and treatment modalities primarily revolve around hospital visits. This has resulted in the accumulation of many patients in a small number of tertiary hospitals with high-quality medical resources for offline diagnosis and treatment [57]. Consequently, personal active health management is rarely implemented. However, hierarchical diagnosis and treatment systems and family doctor consultation models have been primarily implemented in American countries [58]. In the American context, family doctors, who constitute around 80% of doctors, are responsible for 80%-90% of primary diagnosis and treatment services [59]. These services primarily entail disease prevention through active health management. Contrastingly, China relies predominantly on traditional hospitals and drug payments. As a result, Chinese hospitals primarily prioritize in-hospital drug efficacy and often overlook out-of-hospital patient management. In addition, limited software copyright protection in China restricts profits for HMA developers from software downloads. Consequently, the substantial economic costs have impeded the development of HMAs in China. However, American countries primarily implement medical value–based payment policies. Medical insurance payers prioritize patient rehospitalization rates, imposing fines on medical institutions for frequent patient rehospitalizations. This has prompted American medical institutions to use digital health apps to manage patients after discharge and monitor their health status in real-time, aiming to reduce rehospitalization rates. Furthermore, in the United States, HMAs can be prescribed to patients by family doctors or specialists through digital therapy prescriptions. Moreover, the software is granted patent rights, and app developers can cover app development–related costs through software downloads or paid functions, thereby promoting the continuous upgrading and iteration of HMAs.

### Satisfaction and Focus of Chinese and American MHA Users

The overall user satisfaction with HMAs was generally poor, with Chinese HMAs exhibiting higher user satisfaction compared with American HMAs. The functions, software design, technical stability, and cost of HMAs were common areas of concern for both Chinese and American users, although their specific focuses differed. The disparity in user satisfaction between Chinese and American HMAs was consistent with the topic *clustering results of user reviews*, with only Chinese HMA user reviews containing topics reflecting positive user attitudes. Furthermore, the overall user satisfaction obtained in this study, using big data generated by real-world users, was lower than that reported by previous small-sample surveys [60] (87,773/116,686, 75.22%, vs 93.5%). Nevertheless, HMA user engagement and intention to continue use were low [19,20], indicating that our study results are more reliable than those of previous small-sample questionnaire surveys.

Regarding qualitative user descriptions, both Chinese and American users expressed concerns about hypertension management functions, software design, technical stability, and costs. Specifically, most users were particularly concerned about hypertension management functions such as BP tracking and heart rate detection, including BP and heart rate measurements, BP recording, and the visual display of BP change trends. Software design, encompassing usability and interface design, was also a primary concern among users. Usability emerged as the topic of greatest concern among both Chinese (6863/16,561, 41.44%) and American (34,443/100,125, 34.40%) users. Attributes such as simple operating procedures, convenient usage environments, excellent interface design, and clear information display were highlighted as factors contributing to improved user satisfaction. Discussions on technical stability primarily revolved around software reliability, including issues such as software crashes and the inability to open software, as well as system compatibility problems such as software version mismatches and difficulties connecting via Bluetooth. In addition, concerns were raised about measurement accuracy. Finally, users also expressed concerns about software costs, including whether the software is free, its affordability, and the possibility of refunds.

However, there was a significant difference in the focus of HMAs between Chinese and American user groups. Chinese users primarily paid attention to technological stability, such as software reliability and measurement accuracy. By contrast, American users were more concerned about specific hypertension management functions. Moreover, Chinese and American users exhibited different concerns regarding software functions and design. Chinese users showed more interest in the real-time software monitoring function, while American users focused more on data synchronization, data sharing, and BP management effects. Furthermore, Chinese users mentioned software design and personalization, whereas American users were more inclined to allow advertisements that did not disrupt normal app use.

### Motivation and Hygiene Factors of Chinese and American HMAs

Easy-to-use features and interface design, including simple software operation and convenience, were common motivational factors for both Chinese and American HMA users. Compatibility issues, such as mismatched software versions and the inability to connect via Bluetooth, were common hygienic factors. However, other motivational and hygienic factors differed considerably between the 2 user groups. Tobit model analysis and the Wald test revealed influencing factors with significantly different effects on user satisfaction PD and ND, indicating asymmetric impacts of related factors on Chinese and American HMA user satisfaction. This suggests the presence of both motivational and hygienic factors in influencing user satisfaction.

Simple operating procedures and convenient HMA usage effectively improve user satisfaction. However, other motivational factors had significantly different effects on user satisfaction. In addition to the motivational factors for Chinese users, American users prioritized factors such as the BP tracking function, BP management effect, measurement accuracy, and cost-effectiveness. The presence of these influencing factors in user reviews increased the degree of user satisfaction, while their absence had the opposite effect. Hence, these factors were identified as the main contributors to user satisfaction, although user dissatisfaction was less associated with them. Therefore, according to the 2-factor theory, the aforementioned influencing factors were considered motivational factors for HMA user satisfaction. Enhancing these factors can lead to increased user satisfaction and, consequently, strengthen users’ intention to continue using the HMA.

Addressing issues such as software version mismatches and the inability to connect via Bluetooth can effectively mitigate user dissatisfaction. However, other hygienic factors had varying effects on user satisfaction. In addition to measurement accuracy and cost, hygienic factors for Chinese users encompassed aspects such as data sharing (ie, data uploadable to family doctors). By contrast, hygienic factors for American users included reliability, data synchronization, advertisements that did not affect normal use, and heart rate detection functions. The presence of the aforementioned influencing factors in user reviews increased the degree of user dissatisfaction, and vice versa. Hence, while users feel dissatisfied when these factors do not meet their expectations, their satisfaction is not significantly affected. Therefore, according to the 2-factor theory, the aforementioned influencing factors were considered hygienic factors for HMA user satisfaction. Improving these factors can help mitigate user dissatisfaction, thereby enhancing user participation.

### Suggestions for Improving User Engagement and Continued Use of HMA

Given the considerable differences in motivational and hygienic factors for HMA use between Chinese and Americans, software developers should tailor improvements to hygienic factors based on the specific needs of each user group. This approach can effectively reduce user dissatisfaction and increase user participation. Furthermore, efforts should be directed toward enhancing motivational factors to improve user satisfaction and foster continued use intention among both Chinese and American users. Considering that hygiene factors have a direct impact on the utilization of HMA, it is advisable for developers to prioritize improving these factors to enhance user participation. Once hygiene factors are satisfactorily addressed, developers can then focus on enhancing motivational factors to increase users’ willingness to continue using the HMA. By improving corresponding motivational and hygienic factors tailored to different user groups, developers can facilitate the adoption and utilization of HMA, thereby aiding patients in effectively controlling BP. For Chinese HMAs, developers should prioritize enhancing the accuracy of indicator measurements by improving the sensitivity of measurement sensors and optimizing software measurement algorithms. This targeted improvement can effectively mitigate user abandonment of Chinese HMAs. In addition, HMA developers should promptly address technical issues related to software compatibility, such as mismatches between phone systems and software versions, as well as Bluetooth connectivity issues. Moreover, given that Chinese users are more price-sensitive, high software charges have resulted in the loss of a significant number of users. Therefore, developers should consider implementing pricing strategies that align with the preferences and financial capabilities of Chinese users to mitigate user attrition. Developers should consider reducing the number of charging items, shifting away from the traditional model of charging for software downloads, and exploring new business models to optimize profits. For instance, HMAs could be bundled with BP monitors or other relevant health devices to provide added value to users. Furthermore, government intervention is crucial to support the growth of digital medical companies and provide funding for the development of mHealth solutions, especially those focused on chronic disease management. Policy support can encourage entrepreneurship in the digital health sector and foster innovation in HMA development. Finally, HMA developers can enhance user satisfaction by focusing on improving app usability and interface design. This could involve streamlining the operation process, providing clear operation guides, and enhancing the overall user-friendliness of the software. By prioritizing these aspects, developers can create a more intuitive and enjoyable user experience, ultimately increasing user satisfaction and engagement with the HMA.

For American HMAs, developers should promptly address software compatibility issues to enhance HMA usage. This includes resolving mismatches between wearable devices and software, as well as fixing Bluetooth connection failures. In addition, HMA developers should consider incorporating health data sharing and export functions to fulfill the fundamental requirements of American users. Furthermore, developers should optimize HMA reliability and promptly address technical issues, such as software crashes and start-up failures. In addition, providing data synchronization functionality for wearable device monitoring software is essential to ensure real-time BP tracking. Furthermore, developers should consider developing additional heart rate detection functionality to meet users’ needs for tracking heart rate indicators. In addition, HMA developers should reconsider the placement and frequency of advertisements to avoid disrupting routine HMA use. Finally, in addition to enhancing the satisfaction of American HMA users, developers can improve the effectiveness of BP management within the software, enhance indicator measurement accuracy, optimize interface design, and establish a reasonable charging model. For instance, they could refine BP measurement algorithms and validate measurement results against those obtained from a BP monitor. They can incorporate additional BP management features such as exercise and diet management, BP warnings, and visual displays of BP trends. Enhancing the interface aesthetics and integrating HMAs into medical insurance schemes are also crucial measures to consider.

### Advantages Compared With Previous Research

This study marks a significant advancement by exploring user satisfaction and its influencing factors on HMAs based on real-world user reviews. Unlike previous research that primarily relied on on-site surveys and qualitative analysis, often gathering subjective comments through interviews or questionnaires, this study leverages real user feedback obtained from online reviews. By using advanced computational methods such as natural language processing and topic modeling, it provides a more comprehensive and data-driven analysis of user satisfaction factors. For instance, Breil et al [61] discovered, through questionnaire surveys, that both patients and doctors generally accept HMAs, with expected performance being a crucial determining factor. Similarly, Kang et al [30] developed an HMA and assessed user satisfaction using a scale. However, our study adopts a big data–driven approach to quantitatively explore the influencing factors of user satisfaction in HMAs. Using the LDA model, we mined user perspectives from user-generated content and established a connection between user perspectives and satisfaction through the Tobit model, rendering the results more objective and reliable. For practical applications, previous studies have primarily focused on user groups in specific regions, overlooking the exploration of user satisfaction asymmetry. For instance, Hui et al [62] evaluated the functional availability and user satisfaction of HMAs in the United Kingdom, highlighting the need for further improvement. Meanwhile, Melin et al [63] developed an app user satisfaction evaluation scale and assessed app user satisfaction using linear weighting methods. This study compared user satisfaction and influencing factors of HMAs in China and the United States, applying the 2-factor theory to analyze user satisfaction asymmetry. This provided deeper insights into the attributes of influencing factors and offered more accurate improvement suggestions.

### Limitations

This study had several limitations. First, due to variations in medical policies, diagnoses, and treatment models between China and the United States, Chinese individuals use HMAs less frequently, resulting in a proportionately lower number of Chinese reviews and downloads (16,561/24,204,832, 0.07%) compared with American ones (100,125/148,869,181, 0.07%). Therefore, the number of collected Chinese HMA user reviews was significantly lower than that for the American apps. However, for the representativeness of user reviews, we did not conduct special sampling but obtained all user reviews. These user reviews were deemed sufficient to reflect user satisfaction and the influencing factors. Second, considering that some users used the HMA but did not leave reviews, there may have been bias in population selection, and we were unable to explore the satisfaction of such users. However, given that 116,686 reviews were included, and the process of user reviews is random, with users of various opinions possibly not leaving reviews, we believe our results are representative and provide useful data for discovering factors and attributes associated with HMA user satisfaction. Third, through user reviews, this study identified the factors influencing user satisfaction and explored their asymmetry. However, an in-depth analysis of the impact paths could be further explored based on these influencing factors. Finally, through data screening and aggregation, we observed that the number of Chinese HMA user reviews was substantially lower than that of American HMA user reviews, and the factors affecting user satisfaction in China and the United States were considerably different. Although we provided a preliminary analysis of the reasons for these differences through a sociotechnical model, a more in-depth analysis is required in the future. In addition, as users are more concerned about the therapeutic effect of HMAs, it would be meaningful to pair clinical efficacy data with satisfaction in future research to further explore the relationship between user satisfaction and specific clinical efficacy.

### Conclusions

Our study reveals that only 87,773/116,686 (75.22%) users are satisfied with HMA use. We also found that the factors influencing Chinese and American HMA user satisfaction exhibit asymmetry. Furthermore, because of differences in user groups and macro usage environments, the motivational and hygienic factors for users in China and the United States are significantly different. Thus, to enhance user participation, developers of HMAs should devise personalized and comprehensive strategies that address issues related to hygienic factors as a priority. Furthermore, efforts should be made to enhance motivational factors to encourage sustained HMA usage.

## Appendix

Multimedia Appendix 1 Search terms and results; inclusion and exclusion criteria; annotation and examples of reviews (in both Chinese and English). We also present the results of multicollinearity analysis.

## Abbreviations

BP: blood pressure
FDA: Food and Drug Administration
HIT: health information technology
HMA: hypertension management app
LDA: latent Dirichlet allocation
mHealth: mobile health
ND: negative deviation
PD: positive deviation
PRISMA: Preferred Reporting Items for Systematic Reviews and Meta-Analyses

## Data Availability

We used crawler technology and Qimai app data official website to obtain all hypertension management apps and user reviews in 8 Chinese app stores (China Apple App Store, Huawei App Store, Xiaomi App Store, OPPO App Store, VIVO App Store, Baidu App store, 360 App store, and Application Treasure App Store) and 2 American app stores (US Apple App Store and US Google Play Store). The source code for our analysis is publicly available on GitHub [64].
