# Supplementary material for: Mining User Reviews From Hypertension Management Mobile Health Apps to Explore Factors Influencing User Satisfaction and Their Asymmetry: Comparative Study
Source: JMIR Mhealth Uhealth. 2024 Mar 28;12:e55199. doi: 10.2196/55199 (PMC11009850; doi:10.2196/55199)
Supplement: Multimedia Appendix 1 [file mhealth_v12i1e55199_app1.docx]

# Multimedia Appendix 1

**For Manuscript:**

**"Mining User Reviews From Hypertension Management Mobile Health Apps to Explore User Satisfaction Influencing Factors and Their Asymmetry: Comparative Study"**

# 1 Summary of Tables

Table 1 Search terms and results for Chinese and English App stores

| **App stores** | **Search terms** | **Search results** |
| --- | --- | --- |
| **Chinese App Store** | | |
| China Apple App Store | “血压”, “血压管理”, “血压控制”, “血压监测”, “血压测量”, “血压记录”, “高血压”, “高血压预防”, “高血压治疗”, “高血压随访”, “hypertension”, and “high blood pressure” | 566 |
| Huawei App Store | “血压”, “血压管理”, “血压控制”, “血压监测”, “血压测量”, “血压记录”, “高血压”, “高血压预防”, “高血压治疗”, “高血压随访”, “hypertension”, and “high blood pressure” | 730 |
| Xiaomi App Store | “血压”, “血压管理”, “血压控制”, “血压监测”, “血压测量”, “血压记录”, “高血压”, “高血压预防”, “高血压治疗”, “高血压随访”, “hypertension”, and “high blood pressure” | 475 |
| OPPO App Store | “血压”, “血压管理”, “血压控制”, “血压监测”, “血压测量”, “血压记录”, “高血压”, “高血压预防”, “高血压治疗”, “高血压随访”, “hypertension”, and “high blood pressure” | 463 |
| VIVO App store | “血压”, “血压管理”, “血压控制”, “血压监测”, “血压测量”, “血压记录”, “高血压”, “高血压预防”, “高血压治疗”, “高血压随访”, “hypertension”, and “high blood pressure” | 538 |
| Baidu App store | “血压”, “血压管理”, “血压控制”, “血压监测”, “血压测量”, “血压记录”, “高血压”, “高血压预防”, “高血压治疗”, “高血压随访”, “hypertension”, and “high blood pressure” | 60 |
| 360 App store | “血压”, “血压管理”, “血压控制”, “血压监测”, “血压测量”, “血压记录”, “高血压”, “高血压预防”, “高血压治疗”, “高血压随访”, “hypertension”, and “high blood pressure” | 340 |
| Application treasure App store | “血压”, “血压管理”, “血压控制”, “血压监测”, “血压测量”, “血压记录”, “高血压”, “高血压预防”, “高血压治疗”, “高血压随访”, “hypertension”, and “high blood pressure” | 416 |
| **English App Store** | | |
| US Apple App Store | “hypertension”, and “high blood pressure” | 533 |
| US Google Play Store | “hypertension”, and “high blood pressure” | 895 |

Table 2 Detailed inclusion and exclusion criteria for hypertension management Apps

| **Inclusion/exclusion criteria** | **Standard content** |
| --- | --- |
| Inclusion criterion 1 (IC1) | The search terms were “血压”, “血压管理”, “血压控制”, “血压监测”, “血压测量”, “血压记录”, “高血压”, “高血压预防”, “高血压治疗”, “高血压随访”, “hypertension”, and “high blood pressure” for Chinese App Store and “hypertension”, and “high blood pressure” for English App Store. |
| Inclusion criterion 2 (IC2) | Belonging to either health or medical Apps. |
| Inclusion criterion 3 (IC3) | Must included Apps for the blood pressure management, control, monitoring, measurement, recording, prevention, treatment or follow-up. |
| Inclusion criterion 4 (IC4) | The target users were those who need to control their blood pressure. |
| Exclusion criterion 1 (EC1) | Apps were only data receiving and transferring ends of external facilities or sensors and did not function for the control, monitoring, measurement, recording, prevention, treatment or follow-up of blood pressure. |
| Exclusion criterion 2 (EC2) | Apps have been removed from the App store. |
| Exclusion criterion 3 (EC3) | Blood pressure management is not the main function. |
| Exclusion criterion 4 (EC4) | The language used by the Apps is not Chinese or English. |
| Exclusion criterion 5 (EC5) | Apps for no user review. |

Table 3 Keywords and examples of information annotation

| **Chinese reviews** | | **English reviews** | |
| --- | --- | --- | --- |
| **Keywords** | **Examples of annotated text** | **Keywords** | **Examples of annotated text** |
| 测量准确度 | 2分钟前156-101， 一分钟之后132-83 | accuracy | 140/78 |
| 费用 | 花了12块钱 | fee | $49/ year |
| 软件版本 | 1.6新版本用不了 | software version | 1.83 version |
| 综合质量 | 100分，非常好 | overall quality | 5 star Great |
| 使用次数 | 用了不到10次 | frequency | 3 times |
| 使用日期 | 2012年购买并使用 | used date | 3/2022 |
| 测量时长 | 10秒就测出来了 | measure time | within 5 seconds |
| 使用时长 | 用了1年多 | usage time | 2 years |
| 软件大小 | 软件居然占180M内存 | software size | 55MB |
| 使用设备 | 小米手环3 | use device | Omron device |

Table 4 Examples of reviews for each topic in Chinese reviews

| **Topics** | **Reviews (example)** |
| --- | --- |
| Topic 1: easy to use | 很好用的一个APP，用起来很方便，操作简单易上手 |
| Topic 2: reliability | 下载后登陆不了，还频繁闪退，很无语 |
| Topic 3: measurement accuracy | 测量结果很准确，测量数据跟我在医院测量的结果差不多 |
| Topic 4: attitude (positive) | 很好，用过这么多还是这款最好，很好的构思。我非常喜欢，推荐！大家可以试试 |
| Topic 5: compatibility | 小米外的安卓手机安装后无法打开，兼容性特别差 |
| Topic 6: cost | 突然弹出一个408元的内购，巨坑。不要下这个软件，进去随便看看就直接弹个408元的订阅付费，还是每年自动续费的，这算是恶意诱导用户付费 |
| Topic 7: heart rate detection function | 血压和心率测量都有，心率测量有图表追踪，很好的功能 |
| Topic 8: blood pressure tracking function | 记录血压很方便。比较喜欢里面的血压记录功能，不需要每次测量血压后手抄到本子上了 |
| Topic 9: interface design | 界面简洁美观，使用方便，可以随意添加/隐藏各种小部件。 |
| Topic 10: real-time | 随时随地都可以测量并记录血压，很方便 |
| Topic 11: data privacy | 需要确保个人的隐私数据不泄露 |

Table 5 Examples of reviews for each topic in English reviews

| **Topics** | **Reviews (example)** |
| --- | --- |
| Topic 1: easy to use | A great app. Simple to use & easy to read! |
| Topic 2: blood pressure tracking function | Blood pressure checker history app is amazing app to make your Blood pressure record. Recommended! |
| Topic 3: data synchronization | synchronization doesn't work well and app runs all the time. also doesn't allow to synch to other apps to store data in one place |
| Topic 4: blood pressure management effect | Helped lower blood pressure just by monitoring it. |
| Topic 5: heart rate detection function | A great app that makes it easy for anyone to use this app to measure their heart rate and blood pressure and an amazing experience for sure. |
| Topic 6: data sharing | Great BP monitor. I have found the Quardio to be an excellent and accurate health support device. I wish there was more data export ability function however. |
| Topic 7: reliability | The app gave me problems, and now my problem is the app emails the file in a format not able to open. The file is called Sdcard. Not recognized. And cannot be opened. |
| Topic 8: compatibility | Does not work on iPhone 13 pro max. 3 cameras does not allow the app to work. |
| Topic 9: interface design | A good program with plenty of options and good interface. |
| Topic 10: advertisement distribution | Too many advertisements, all advertisements |
| Topic 11: measurement accuracy | A good way to track your blood pressure with accuracy. |
| Topic 12: cost | App price. Anubis this app a subscription based app after laying out some good ones for the product? $70 plus another $100 minimum per year? This is too expensive. |

Table 6 Explanation of each topic in Chinese reviews

| **Topics** | **Interpretation of the topic** |
| --- | --- |
| Topic 1: easy to use | It refers to the simple and convenient design of App functions, which users can quickly get started with. |
| Topic 2: reliability | It refers to the stability of the App's operation, whether there are problems such as inability to open, frequent crashes, and inability to use. |
| Topic 3: measurement accuracy | It refers to the accuracy of the App in measuring indicators such as blood pressure and heart rate, and whether they are consistent with the values measured in the consultation room. |
| Topic 4: attitude (positive) | It refers to the positive emotions of users after using the App, as well as their love for the App. |
| Topic 5: compatibility | It refers to the compatibility between the App and the mobile phone or operating system, and whether there are compatibility issues with the mobile phone or operating system after downloading. |
| Topic 6: cost | It refers to the amount of money spent by users downloading or using the App. |
| Topic 7: heart rate detection function | It refers to the detection of user heart rate by the App, measuring and recording the user's heart rate value. |
| Topic 8: blood pressure tracking function | It refers to the monitoring of user blood pressure by the App, including measuring blood pressure, recording blood pressure, and dynamically displaying changes in blood pressure. |
| Topic 9: interface design | It refers to the interface aesthetics and component design of the App. |
| Topic 10: real-time | It refers to the time integrity and timeliness of App indicator monitoring. |
| Topic 11: data privacy | It refers to the protection of user privacy data by APP. |

Table 7 Explanation of each topic in English reviews

| **Topics** | **Interpretation of the topic** |
| --- | --- |
| Topic 1: easy to use | It refers to the simple and convenient design of App functions, which users can quickly get started with. |
| Topic 2: blood pressure tracking function | It refers to the monitoring of user blood pressure by the App, including measuring blood pressure, recording blood pressure, and dynamically displaying changes in blood pressure. |
| Topic 3: data synchronization | It refers to whether the data measured through blood pressure monitors and wearable devices can be uploaded to the App in a timely manner, and whether the Bluetooth connection for data transmission is stable. |
| Topic 4: blood pressure management effect | It refers to whether the App is helpful for controlling the user's blood pressure. |
| Topic 5: heart rate detection function | It refers to the detection of user heart rate by the App, measuring and recording the user's heart rate value. |
| Topic 6: data sharing | It refers to whether the user data of the App can be shared with others or in the form of reports to doctors. |
| Topic 7: reliability | It refers to the stability of the App's operation, whether there are problems such as inability to open, frequent crashes, and inability to use. |
| Topic 8: compatibility | It refers to the compatibility between the App and the mobile phone or operating system, and whether there are compatibility issues with the mobile phone or operating system after downloading. |
| Topic 9: interface design | It refers to the interface aesthetics and component design of the App. |
| Topic 10: advertisement distribution | It refers to the frequency and location of advertisements appearing in the App, as well as whether advertisements hinder the normal use of functions. |
| Topic 11: measurement accuracy | It refers to the accuracy of the App in measuring indicators such as blood pressure and heart rate, and whether they are consistent with the values measured in the consultation room. |
| Topic 12: cost | It refers to the amount of money spent by users downloading or using the App. |

Table 8 Multicollinearity analysis for model 1

| **Influencing factor** | **Tolerance** | **VIF** |
| --- | --- | --- |
| Topic 1: easy to use | 0.86 | 1.16 |
| Topic 2: reliability | 0.96 | 1.04 |
| Topic 3: measurement accuracy | 0.94 | 1.06 |
| Topic 5: compatibility | 0.97 | 1.03 |
| Topic 6: cost | 0.98 | 1.02 |
| Topic 7: heart rate detection function | 0.98 | 1.02 |
| Topic 8: blood pressure tracking function | 0.98 | 1.02 |
| Topic 9: interface design | 0.99 | 1.01 |
| Topic 10: real-time | 0.99 | 1.01 |
| Topic 11: data privacy | 0.99 | 1.01 |
| Dependent variable: positive rating deviations | | |

Table 9 Multicollinearity analysis for model 2

| **Influencing factor** | **Tolerance** | **VIF** |
| --- | --- | --- |
| Topic 1: easy to use | 0.86 | 1.16 |
| Topic 2: reliability | 0.96 | 1.04 |
| Topic 3: measurement accuracy | 0.94 | 1.06 |
| Topic 5: compatibility | 0.97 | 1.03 |
| Topic 6: cost | 0.98 | 1.02 |
| Topic 7: heart rate detection function | 0.98 | 1.02 |
| Topic 8: blood pressure tracking function | 0.98 | 1.02 |
| Topic 9: interface design | 0.99 | 1.01 |
| Topic 10: real-time | 0.99 | 1.01 |
| Topic 11: data privacy | 0.99 | 1.01 |
| Dependent variable: negative rating deviations | | |

Table 10 Multicollinearity analysis for model 3

| **Influencing factor** | **Tolerance** | **VIF** |
| --- | --- | --- |
| Topic 1: easy to use | 0.82 | 1.22 |
| Topic 2: blood pressure tracking function | 0.85 | 1.17 |
| Topic 3: data synchronization | 0.87 | 1.14 |
| Topic 4: blood pressure management effect | 0.83 | 1.2 |
| Topic 5: heart rate detection function | 0.95 | 1.06 |
| Topic 6: data sharing | 0.98 | 1.02 |
| Topic 7: reliability | 0.86 | 1.16 |
| Topic 8: compatibility | 0.96 | 1.04 |
| Topic 9: interface design | 0.99 | 1.01 |
| Topic 10: advertisement distribution | 0.98 | 1.02 |
| Topic 11: measurement accuracy | 0.98 | 1.02 |
| Topic 12: cost | 0.99 | 1.01 |
| Dependent variable: positive rating deviations | | |

Table 11 Multicollinearity analysis for model 4

| **Influencing factor** | **Tolerance** | **VIF** |
| --- | --- | --- |
| Topic 1: easy to use | 0.82 | 1.22 |
| Topic 2: blood pressure tracking function | 0.85 | 1.17 |
| Topic 3: data synchronization | 0.87 | 1.14 |
| Topic 4: blood pressure management effect | 0.83 | 1.2 |
| Topic 5: heart rate detection function | 0.95 | 1.06 |
| Topic 6: data sharing | 0.98 | 1.02 |
| Topic 7: reliability | 0.86 | 1.16 |
| Topic 8: compatibility | 0.96 | 1.04 |
| Topic 9: interface design | 0.99 | 1.01 |
| Topic 10: advertisement distribution | 0.98 | 1.02 |
| Topic 11: measurement accuracy | 0.98 | 1.02 |
| Topic 12: cost | 0.99 | 1.01 |
| Dependent variable: negative rating deviations | | |

Table 12 Comparison between the parameters of models 1 and 2 (Wald test)

| **Influencing factor** | **Wald test** | **P value** | **n in PD(%)** | **n in ND(%)** |
| --- | --- | --- | --- | --- |
| Topic 1: easy to use | 207.64 | <0.001 | 6114 (89.09%) | 749 (10.91%) |
| Topic 2: reliability | 30.45 | <0.001 | 1809 (83.56%) | 356 (16.44%) |
| Topic 3: measurement accuracy | 682.27 | <0.001 | 1294 (61.88%) | 797 (38.12%) |
| Topic 5: compatibility | 700.55 | <0.001 | 388 (44.19%) | 490 (55.81%) |
| Topic 6: cost | 909.11 | <0.001 | 281 (43.03%) | 372 (56.97%) |
| Topic 7: heart rate detection function | 0.00 | 0.981 | 499 (83.58%) | 98 (16.42%) |
| Topic 8: blood pressure tracking function | 0.48 | 0.490 | 408 (81.27%) | 94 (18.73%) |
| Topic 9: interface design | 75.48 | <0.001 | 331 (70.28%) | 140 (29.72%) |
| Topic 10: real-time | 55.59 | <0.001 | 199 (66.56%) | 100 (33.44%) |
| Topic 11: data privacy | 11.57 | 0.001 | 142 (73.96%) | 50 (26.04%) |

PD: positive deviation. ND: negative deviation.

Table 13 Comparison between the parameters of models 3 and 4 (Wald test)

| **Influencing factor** | **Wald test** | **P value** | **n in PD(%)** | **n in ND(%)** |
| --- | --- | --- | --- | --- |
| Topic 1: easy to use | 1537.23 | <0.001 | 27088 (78.65%) | 7355 (21.35%) |
| Topic 2: blood pressure tracking function | 959.82 | <0.001 | 14428 (83.47%) | 2857 (16.53%) |
| Topic 3: data synchronization | 11766.07 | <0.001 | 2754 (21.45%) | 10083 (78.55%) |
| Topic 4: blood pressure management effect | 3126.46 | <0.001 | 10338 (87.01%) | 1543 (12.99%) |
| Topic 5: heart rate detection function | 568.70 | <0.001 | 4142 (58.63%) | 2923 (41.37%) |
| Topic 6: data sharing | 396.61 | <0.001 | 2322 (52.00%) | 2143 (48.00%) |
| Topic 7: reliability | 1696.82 | <0.001 | 1129 (29.77%) | 2664 (70.23%) |
| Topic 8: compatibility | 1754.99 | <0.001 | 1116 (43.16%) | 1470 (56.84%) |
| Topic 9: interface design | 624.08 | <0.001 | 2044 (85.52%) | 346 (14.48%) |
| Topic 10: advertisement distribution | 4960.95 | <0.001 | 329 (20.58%) | 1270 (79.42%) |
| Topic 11: measurement accuracy | 127.02 | <0.001 | 926 (77.23%) | 273 (22.77%) |
| Topic 12: cost | 10.91 | 0.001 | 471 (80.93%) | 111 (19.07%) |

PD: positive deviation. ND: negative deviation.

# 2 Summary of Figure


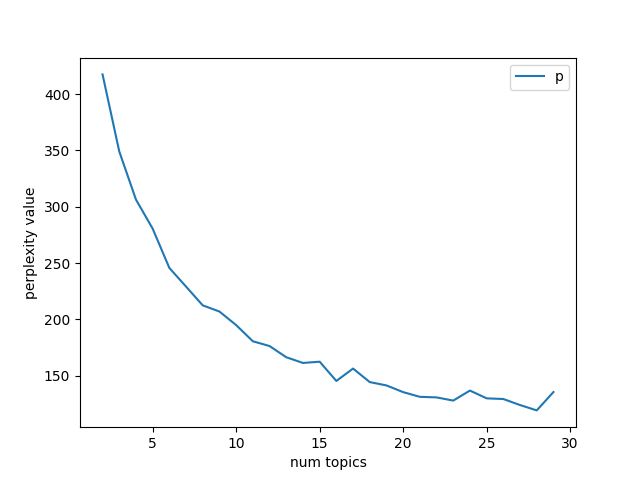


Figure 1 Topics perplexity score (rang between 2 and 29 topics) of Chinese reviews. num: number.


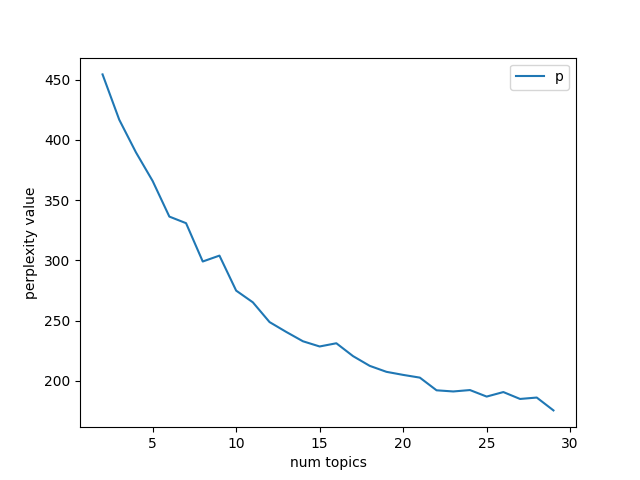


Figure 2 Topics perplexity score (rang between 2 and 29 topics) of English reviews. num: number.


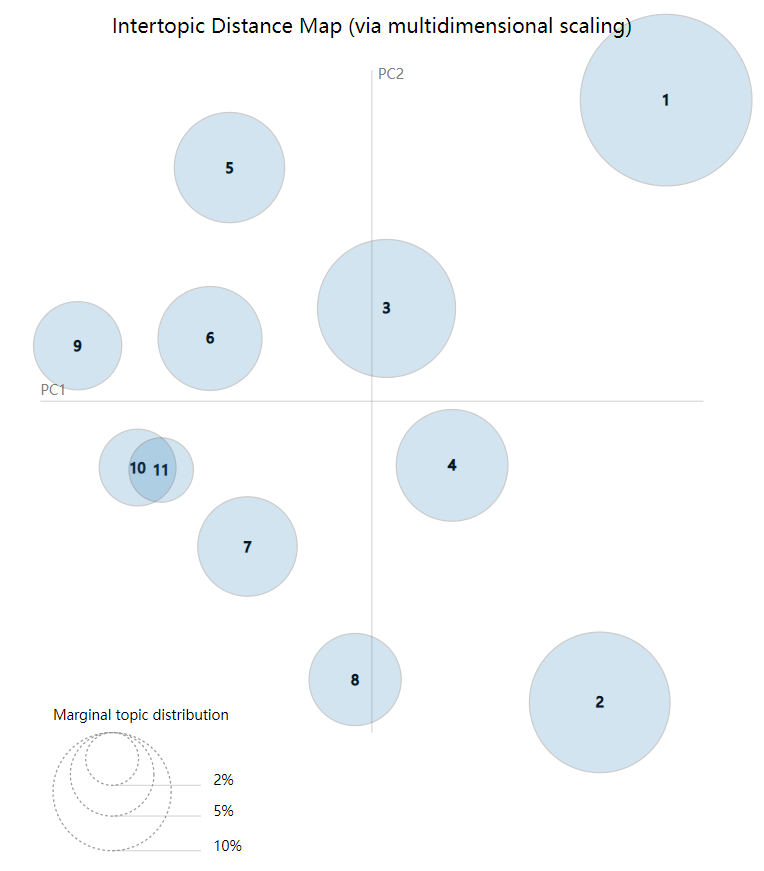


Figure 3 Overview of the formulation of 11 topics in Chinese reviews with LDA topic modeling

Note: Cluster 1: easy to use, Cluster 2: reliability, Cluster 3: measurement accuracy, Cluster 4: compatibility, Cluster 5: cost, Cluster 6: heart rate detection function, Cluster 7: interface design, Cluster 8: real-time, Cluster 9: attitude (positive), Cluster 10: blood pressure tracking function, Cluster 11: data privacy.


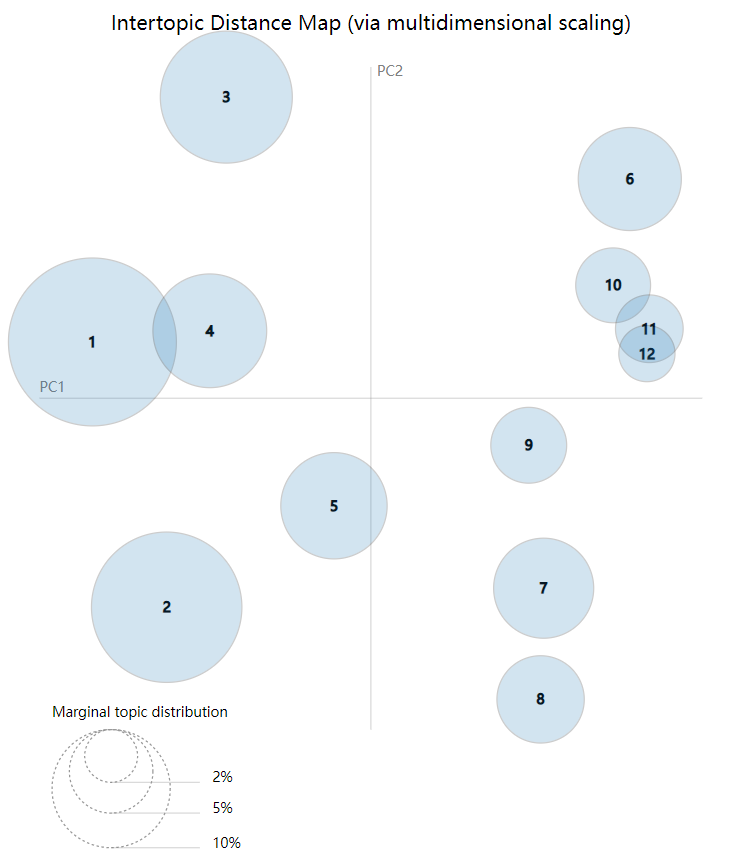


Figure 4 Overview of the formulation of 12 topics in English reviews with LDA topic modeling

Note: Cluster 1: easy to use, Cluster 2: blood pressure tracking function, Cluster 3: blood pressure management effect, Cluster 4: data sharing, Cluster 5: reliability, Cluster 6: data synchronization, Cluster 7: compatibility, Cluster 8: advertisement distribution, Cluster 9: heart rate detection function, Cluster 10: measurement accuracy, Cluster 11: interface design, Cluster 12: cost.

**3 The formula of LDA topic model**

The formulas for PD and ND are as follows:

$$\boldsymbol{PD}_{\boldsymbol{i}}\mathbf{=}\sum_{\boldsymbol{K}\mathbf{=1}}^{\boldsymbol{K}} \boldsymbol{\beta}_{\boldsymbol{PD}\mathbf{,}\boldsymbol{k}} \boldsymbol{\chi}_{\boldsymbol{ki}}\mathbf{+}\boldsymbol{\delta}_{\boldsymbol{PD}\mathbf{,}\boldsymbol{i}}$$

$$\boldsymbol{ND}_{\boldsymbol{i}}\mathbf{=}\sum_{\boldsymbol{K}\mathbf{=1}}^{\boldsymbol{K}} \boldsymbol{\beta}_{\boldsymbol{ND}\mathbf{,}\boldsymbol{k}} \boldsymbol{\chi}_{\boldsymbol{ki}}\mathbf{+}\boldsymbol{\delta}_{\boldsymbol{ND}\mathbf{,}\boldsymbol{i}}$$

where, $\beta_{k}$ is the correlation coefficient between the *k*th topic of review *i* and user satisfaction (${PD}_{i}$ and ${ND}_{i}$), *k* is the number of topics included in the model, and $\delta_{i}$ is the error term.
